# The mitigative effect of ovotransferrin-derived peptide IQW on DSS-induced colitis via alleviating intestinal injury and reprogramming intestinal microbes

**DOI:** 10.3389/fnut.2022.927363

**Published:** 2022-09-02

**Authors:** Yajuan Chai, Sujuan Ding, Lihong Jiang, Shuangshuang Wang, Xiangnan Yuan, Hongmei Jiang, Jun Fang

**Affiliations:** ^1^College of Bioscience and Biotechnology, Hunan Agricultural University, Changsha, China; ^2^Department of Cardiology, Wenling First People's Hospital (The Affiliated Wenling Hospital of Wenzhou Medical University), Wenling, China; ^3^Department of Rehabilitation, Shengjing Hospital of China Medical University, Shenyang, China

**Keywords:** Ile-Gln-Trp, colitis, sodium dextran sulfate, intestinal flora, short-chain fatty acids (SCFA)

## Abstract

Inflammatory bowel disease (IBD) is a chronic disease with multiple complications during its development, and it is difficult to cure. The aim of this study was to evaluate the alleviating effect of different concentrations of the bioactive peptide IQW (Ile-Gln-Trp) on dextran sodium sulfate (DSS)-induced colitis in mice. For this study, we randomly divided 56 ICR mice into seven groups: the (I) control (CON), (II) dextran sodium sulfate treatment (2.5% DSS), (III) IQW-DSS (20 μg/ml) treatment, (IV) IQW-DSS (40 μg/ml) treatment, (V) IQW-DSS (60 μg/ml) treatment, (VI) IQW-DSS (80 μg/ml) treatment, and (VII) IQW-DSS (100 μg/ml) groups. The results showed that IQW at 60 μg/ml alleviated body weight loss, improved the liver index (*p* < 0.05), and improved histomorphological and pathological changes in the colon compared to the DSS-treated group. IQW at 60 μg/ml and IQW at 80 μg/ml modified intestinal microbial disorders. In addition, IQW at 60 μg/ml significantly increased butyric acid levels and decreased valeric acid levels, while IQW at 80 μg/ml significantly increased isobutyric acid and isovaleric acid levels. Hence, IQW at a concentration of 60 μg/ml alleviates DSS-induced colitis by enhancing the body's anti-inflammatory ability and regulating intestinal flora and metabolic changes. In the above context, IQW at 60 μg/ml could be a potential candidate for IBD prevention and treatment.

## Introduction

The global prevalence of IBD, a complex inflammatory disease of the gastrointestinal tract, continues to grow (1, 2). Its main forms include two different chronic recurrent inflammatory diseases, Crohn's disease (CD) and ulcerative colitis (UC) (3). The inflammation of the intestine caused by CD has chronic intermittent symptoms and can affect all segments of the bowel, most commonly the terminal ileum and colon, showing segmental, asymmetrical, and transmural aspects with a variety of complications (4). UC is an inflammatory disease confined to the colonic mucosa, with the disease usually extending from the rectum to the proximal colon and showing alternating symptoms of deterioration and remission (5). The etiology of IBD is unclear, but previous studies have demonstrated that the pathogenesis of IBD is driven by genetic, environmental, immune, and intestinal flora factors (1). Dysbiosis of the gut flora, tissue damage, and metabolic abnormalities are all common symptoms of IBD.

In a healthy state, homeostasis of gut microbes plays an important role in maintaining the host's metabolic balance, immune response, and other physiological stability (6), while during IBD, gut microbial dysbiosis is thought to be partly responsible for IBD, and the composition of the microbiota can regulate the balance between inflammation and homeostasis in the gut (7). Firmicutes show a decrease in abundance, and Proteobacteria show an increase in abundance in the presence of IBD. Some pathogens also show an increase in abundance, such as adherent invasive *Escherichia coli* (*E. coli*) (8, 9). Moreover, bile acid derivatives, short-chain fatty acids (SCFAs), and tryptophan metabolites, all of which are produced from microbial metabolisms, have been linked to gut immunological and inflammatory damages (10). SCFAs, i.e., can be a source of energy for the intestinal epithelium and have an anti-inflammatory activity, which is important for maintaining intestinal homeostasis and maintaining the intestinal barrier and has an impact on changes in the host's body mass index during IBD (11). Hence, prevention and treatment of colitis are particularly important by regulation of changes in gut microbes and their metabolites.

Bioactive peptides are short chains of biologically active amino acids that regulate immune, cardiovascular, neurological, and gastrointestinal physiological responses (12), and they are recognized as an excellent candidate in the development of drugs for treatment of IBD (13). Research has shown that an ovotransferrin-derived peptide, which is the ovalbumin extracted from eggs, has antioxidant activity and antiviral and immunological effects, and it has been used in treatment of diseases (14, 15). Ma Yong et al. found that addition of IQW to diet helped to regulate serum amino acid levels and the expression of inflammatory factors, and to reduce damage to the intestinal barrier (16). Liu et al. investigated the use of bioactive peptides made from egg whites to improve antioxidant effects and maintain intestinal flora balance during treatment of DSS-induced colitis (17). Yutaro Kobayashi et al.'s study showed that oral egg white ovotransferrin can be used as a preventive drug against IBD to maintain intestinal health (18). However, there are only a few studies on the efficacy of different concentrations of IQW in the therapy of DSS-induced colitis. Therefore, this study was conducted to identify an effective treatment for IBD by evaluating the effect of different dietary concentrations of the egg bioactive peptide IQW on dextran sodium sulfate (DSS)-induced colitis in mice.

## Materials and methods

### Animal husbandry and sample collection

Six-week-old female C57BL/6J mice were housed in a rearing room at a room temperature of 22 ± 2°C and a light period of 12 h and were free to drink and eat. After 1 week of acclimatization, 56 mice were randomly divided into 7 groups with 8 mice in each, and IQW was dissolved in drinking water at concentration gradients of 20 μg/ml (20 IQW-DSS), 40 μg/ml (40 IQW-DSS), 60 μg/ml (60 IQW-DSS), 80 μg/ml (80 IQW-DSS), and 100 μg/ml (100 IQW-DSS) for 1 week. The control group and DSS-induced model group were fed pure water for 1 week. The drinking water was then changed to 2.5% DSS (DSS:water/2.5:100) (Mw: 36,000–50,000 Da) for 1 week for the DSS group and the IQW feeding group at different concentrations. The mice were induced with colitis and killed after 3 days of normal feeding with drinking water. Colon tissues and contents were collected to detect colon damage, intestinal microbes, and short-chain fatty acid content in feces. All the experimental animal protocols comply with Chinese animal welfare guidelines. The Hunan Agricultural University Animal Care and Use Committee approved the experiments.

### Immune organ index determination

On the 24th day, the mice were killed. The liver and spleen were dissected and weighed to calculate the immune organ index.

The calculation formula of the immune organ index is as follows:


$$\begin{array}{l} \text{Immune organ index }=\frac{\text{Target organ weight }\left(\text{g}\right)}{\text{Body weight }\left(\text{kg}\right)} \end{array}$$

### Hemoglobin level determination

Wet feces (5 g/100 ml distilled water) were put into a mixer for homogenization, and then 0.5 ml feces homogenates were put into a centrifuge tube for 10 min in a boiling water bath to inactivate the plant oxidase. After the sample was cooled, 30 ml of 30% acetic acid was added and mixed evenly; the above mixture was left to stand for two minutes and then 4.5 ml of ethyl acetate was added and shaken gently for 2 min, and centrifuge at 2000 rmp for 3 min to separate the organic phase and the aqueous phase. One ml of the upper solution was removed, 1 ml of TMB (tetramethylaniline) working solution was added and was mixed well; 0.5 mL of 30 ml /L H_2_O_2_ solution was added and was mixed to start the oxidize the reaction.

We observed the absorbance at A660 for 1 min and recorded it at 30 and 60 s to calculate the hemoglobin content. The hemoglobin standard curve (0.1–1 mg HB) was used to calculate the concentration of fecal hemoglobin with a content of 4–40 mg/g. Samples containing feces with hemoglobin more than 40 mg/g can be diluted with additional distilled water, and the determination results shall be multiplied by the appropriate dilution factor.

The hemoglobin content calculation formula is as follows:


$$\begin{array}{l} \frac{\text{mg Hb}}{\text{g feces}}=\frac{\left[\text{mg Hb}\left(30\text{ s}\right)+\text{mg Hb}\left(60\text{ s}\right)\right]/2}{0.025\text{ g feces}} \end{array}$$

### Intestinal histopathological analysis

The middle section of the intestine of about 2 cm was taken and fixed in a 10% neutral formalin (4% paraformaldehyde) solution. The contents could be removed by changing the fixative 2–3 times within a week after the sample had been taken. The intestinal fixative was washed off with pre-cooled saline, blotted on filter paper, placed in a 10% neutral formalin solution for 72 h, removed, washed 3 times with alcohol, and dehydrated with a gradient of alcohol (75 → 85 → 95 → 100 → 100% II). A microscopic observation of the hematoxylin-stained tissue was then performed. Histological scores were determined according to the severity of inflammation. Grading criteria included (19): (1) inflammation severity scores 0, 1, 2, and 3, respectively, represent none, mild, moderate, and severe; (2) inflammatory cell infiltration scores 0, 1, 2, and 3, respectively, represent normal, mucosal, submucosal, and transmural infiltration; (3) epithelial lesion scores 0, 1, 2, and 3, respectively, represent integrity, crypt structure deformation, erosion, and ulcer; (4) score of lesion degrees 0, 1, 2, and 3, respectively, represent no lesion, point lesion, multifocal lesion, and diffuse lesion; (5) edema scores of 0, 1, 2, and 3, respectively, represent no edema of the mucosa, mild edema, submucosal edema, and edema of the whole colon wall. The results of the histological analysis of the colon were expressed by the sum of scores obtained at all levels.

### 16S rDNA pyrophosphate sequencing

Total DNA was extracted from the microbial genome of each mouse's intestinal segment using the QIAamp Genome Extraction Kit (Qiagen, Hilden, Germany) according to instructions. DNA purity was determined, and DNA concentration was adjusted. The purified DNA was used as a template for PCR amplification using fused primers of the universal primers 357F 5′-ACTCCTACGGRAGGCAGCAG-3′ and 806R 5′-GGACTACHVGGGTWTCTAAT-3′ in the V3–V4 region of 16S rDNA and some Miseq sequencing primers. The PCR products were detected by 1.2% agarose gel electrophoresis. Samples with good detection results were recovered by 2% agarose gel electrophoresis. The recovered products were used as a template for one-time PCR amplification for 8 cycles. An Illumina platform sequencing connector, a sequencing primer, and a tag sequence were added to both ends of the target fragment. All the PCR products were recovered using an AxyPrepDNA gel recovery kit (AXYGEN), and fluorescence quantification was performed using a FTC-3000TM Real-Time PCR instrument. The library was constructed after being mixed with an equal molar ratio. The Illumina Miseq regent V3 kit was used to complete the sequencing of Illumina Miseq PE300 in Microbiotech (Shanghai) Co., Ltd.

The reads of each sample were assigned to the original data with a barcode, and the effective sequences of each sample were obtained. The Trimmomatic software was used to remove the low-quality sequences at the end of the sequencing. According to the overlap relationship between PE reads, the Flash software was used to splice paired reads into a sequence, and the mothur software was used to control and filter the quality of the sequence. The ambiguous bases, homologous regions of single bases, long and short sequences, and some chimeras generated in the PCR process were removed to obtain the optimized sequences. Operational taxonomic unit (OUT) clustering (UPARSE software) was performed, and the OTU representative sequences were compared with the SILVA 128 database for species information annotation. Based on taxonomic information, a statistical analysis of the community structure was carried out at the phylum, class, order, family, genus, and species classification levels. Based on the above analysis, a series of statistical and visual analyses of community structure and phylogeny was carried out. An alpha diversity analysis was performed using mothur (version 1.33.3) (Chao, Ace, Shannon, Simpson, etc.). Based on the OTU analysis of sequence similarity clustering, coverage, sobs, Chao, ACE, Shannon, Simpson, PD whole tree, and other indexes were calculated to obtain alpha diversity, which was used to reflect the abundance and diversity of microbial communities.

### Short-chain fatty acid determination

Sample pre-treatment: we weighed 1 g of the fresh sample accurately and recorded the mass of each sample (0.5 g of dry sample, take care not to get the sample on the cap as much as possible to avoid shaking it off later) and then added 5 ml of ultra-pure water. (1) The vortex was vibrated for 30 min, stored at 4°C overnight, and then centrifuged for 10 min at 1,000 revolutions. The supernatant was transferred, and 4 ml of ultra-pure water was added to the precipitate. It was then shaken for another 30 min and mixed. After centrifugation, the supernatant was merged into a 10-ml coloration dish for constant volume. (2) It was then shaken and mixed for 30 min and centrifuged for 10 min to the transfer supernatant. Two ml of ultrapure water was added; the mixture was shaken and mixed for 30 min and centrifuged, and then the supernatant was transferred. The previous step was then repeated, the supernatant was combined, and the volume was fixed to a 10-mL cuvette. After achieving a constant volume, the liquid was transferred into a 10-ml centrifuge tube and centrifuged at 12,000 rpm for 15 min before mixing it with the supernatant. The supernatant and 25% metaphosphoric acid were added into a 2-ml centrifuge tube at a volume ratio of 9:1 and mixed well, and it was then let to stand and react at room temperature for 3–4 h. After the standing reaction, the mixture was centrifuged, filtered under 45 μm of a microporous membrane, and added to a machine bottle (above 600 μl) for testing. The chromatographic column was DB-FFAP with a specification of 30 m^*^250 μm^*^0.25 μm. High-purity nitrogen and hydrogen (99.999%) were used for the carrier and auxiliary gases, respectively, with a detector FID temperature of 280°C, an inlet temperature of 250 °C, a split ratio of 50:1, and an injection volume of 1 μl. Programmed temperature: the primary temperature was 60°C, and the temperature was increased to 220°C at a rate of 20°C/min for 1 min.

### Data analysis

The experimental data were collated using Excel 2010, and then a one-way ANOVA and Duncan's test were performed on the differences between the groups using the SPSS 16.0 statistical software. The results were expressed as mean ± SEM (*p* < 0.05) and plotted with Origin 8.0. A Pearson correlation analysis was performed using GraphPad prism 8.4 to determine the relationship between different microorganisms and different short-chain fatty acids in the development of DSS-induced inflammation.

## Results

### Effect of different concentration gradients of IQW on the body weight and immune organ index during the development of colitis induced by DSS

**Figure 1:**
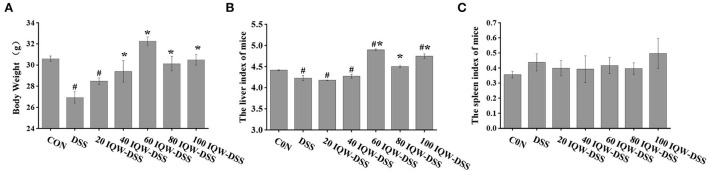
Evaluation of **(A)** body weight and **(B,C)** immune organs affected by egg protein transferrin-derived peptide IQW in the progression of colitis. CON: control group, DSS: mice treated with 2.5% DSS, 20 IQW-DSS: 20 μg/ml IQW and 2.5% DSS-treated mice, 40 IQW-DSS: 40 μg/ml IQW and 2.5% DSS-treated mice, 60 IQW-DSS: 60 μg/ml IQW and 2.5% DSS-treated mice, 80 IQW-DSS: 80 μg/ml IQW and 2.5% DSS-treated mice, and 100 IQW-DSS: 100 μg/ml IQW and 2.5% DSS treated mice; *n* = 8. Note: the results are given as mean ± SEM. ^#^*p* < 0.05 vs. control group, *p < 0.05 vs. the DSS group.

In this study, the evaluation of body weight and immune organs that were affected by egg protein transferrin-derived peptide IQW in the progression of colitis are shown in Figure 1. The results showed that DSS had a negative effect on the body weight of the mice (*p* < 0.05), that IQW (40, 60,80 and 100 μg/ml) could alleviate the effect of DSS on body weight loss (*p* < 0.05), and that 20 μg/ml IQW had no effect on body weight loss (*p* > 0.05) (Figure 1A). Compared with the CON group, the liver weight of the DSS, 20, and 40 μg/ml IQW groups was significantly decreased, while that of the 60 and 100 μg/ml groups was significantly increased (*p* < 0.05). The liver weight of mice in the 60, 80, and 100 μg/ml IQW groups was significantly higher than that of the DSS group (*p* < 0.05) (Figure 1B). There was no significant difference in spleen index among the groups (*p* > 0.05) (Figure 1C).

### Different concentration gradients of IQW treatment can alleviate colitis induced by DSS

**Figure 2:**
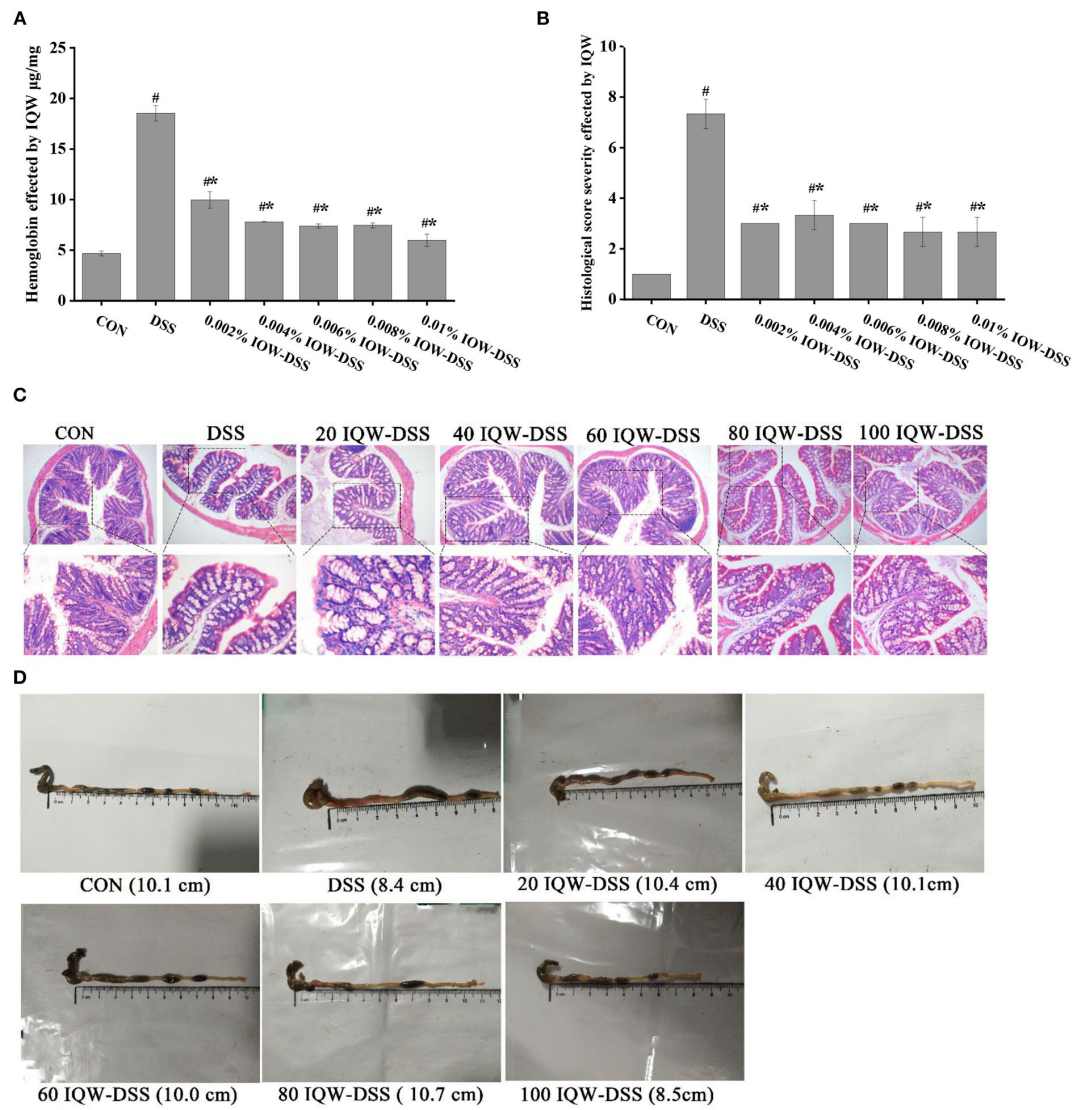
Effects of egg protein transferrin-derived peptide IQW on colitis. **(A)** Hemoglobin levels, **(B)** histological score severity, **(C)** micrograph of H and E-stained colon tissue section (×100 and ×200 magnification), and **(D)** length of the colon. CON: control group, DSS: mice treated with 2.5% DSS, 20 IQW-DSS: 20 μg/ml IQW and 2.5% DSS-treated mice, 40 IQW-DSS: 40 μg/ml IQW and 2.5% DSS-treated mice, 60 IQW-DSS: 60 μg/ml IQW and 2.5% DSS-treated mice, 80 IQW-DSS: 80 μg/ml IQW and 2.5% DSS-treated mice, and 100 IQW-DSS: 100 μg/ml IQW and 2.5% DSS-treated mice. The results are given as mean ± SEM. ^#^*p* < 0.05 vs. the control group, *p < 0.05 vs. the DSS group.

The effects of egg protein transferrin-derived peptides IQW on colitis are shown in Figure 2. The pathological observation of colonic tissues and feces hemoglobin detection revealed that DSS and IQW-DSS treatment resulted in a significant increase in fetal hemoglobin levels and histological scores, and that IQW-DSS treatment with different concentration gradients significantly reduced feces hemoglobin levels and histological scores compared to the DSS-treated group (*p* < 0.05) (Figures 2A,B). In addition, the results of mice colon sections after HE staining showed that DSS caused the colon tissues to have multiple erosive lesions and extensive inflammatory cell infiltration, and that IQW alleviated the degree of colonic erosion (Figure 2C). The colonic length of the mice treated with different concentration gradients of IQW-DSS was also increased compared to the DSS group (Figure 2D).

### Effect of different concentration gradients of egg active peptide IQW on intestinal microorganisms during the development of colitis induced by DSS

**Figure 3:**
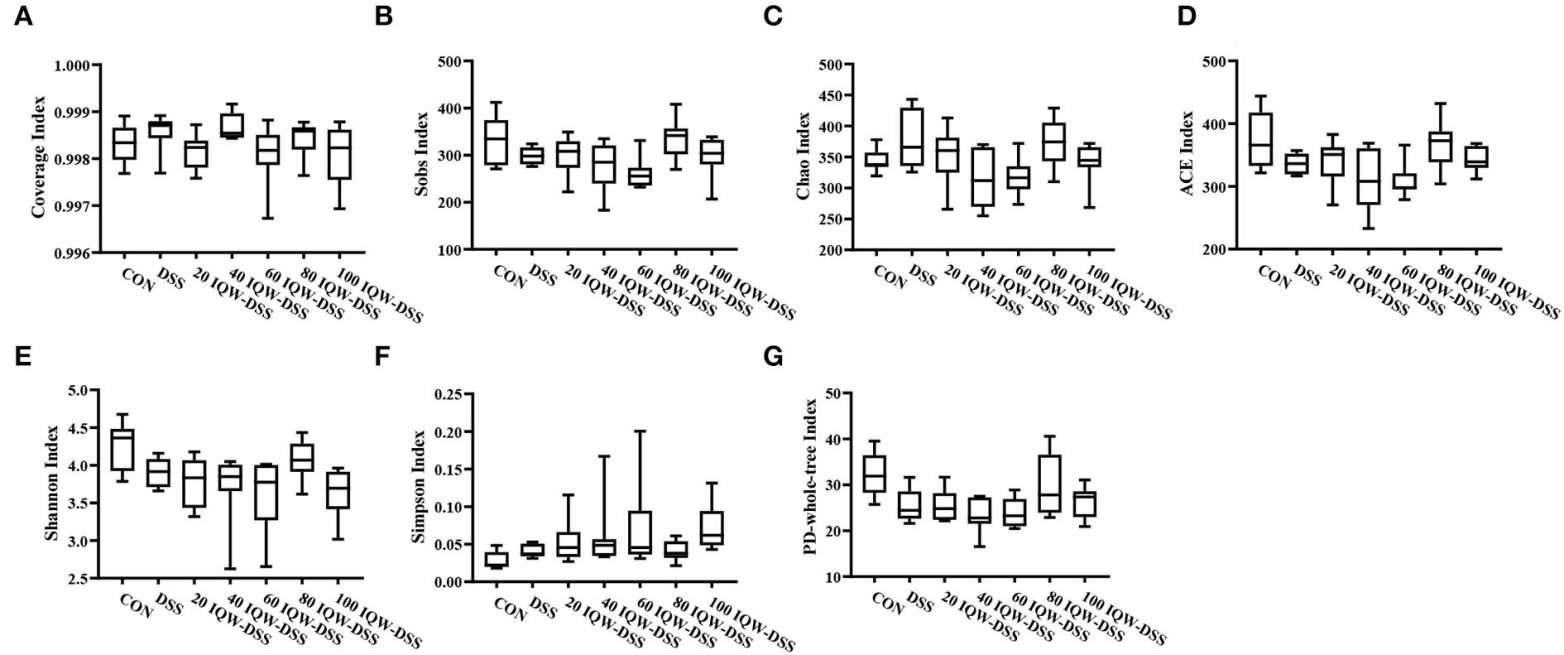
Diversity indexes of microbiota in mouse colon. **(A)** Coverage index, **(B)** Sobs index, **(C)** Chao index, **(D)** ACE index, **(E)** Shannon index, **(F)** Simpson index, and **(G)** PD-whole-tree index. CON1: control group, DSS1: mice treated with 2.5% DSS, 20 IQW-DSS: 20 μg/ml IQW and 2.5% DSS-treated mice, 40 IQW-DSS: 40 μg/ml IQW and 2.5% DSS-treated mice, 60 IQW-DSS: 60 μg/ml IQW and 2.5% DSS-treated mice, 80 IQW-DSS: 80 μg/ml IQW and 2.5% DSS-treated mice, and 100 IQW-DSS1: 100 μg/ml IQW and 2.5% DSS-treated mice; *n* = 8.

**Figure 4:**
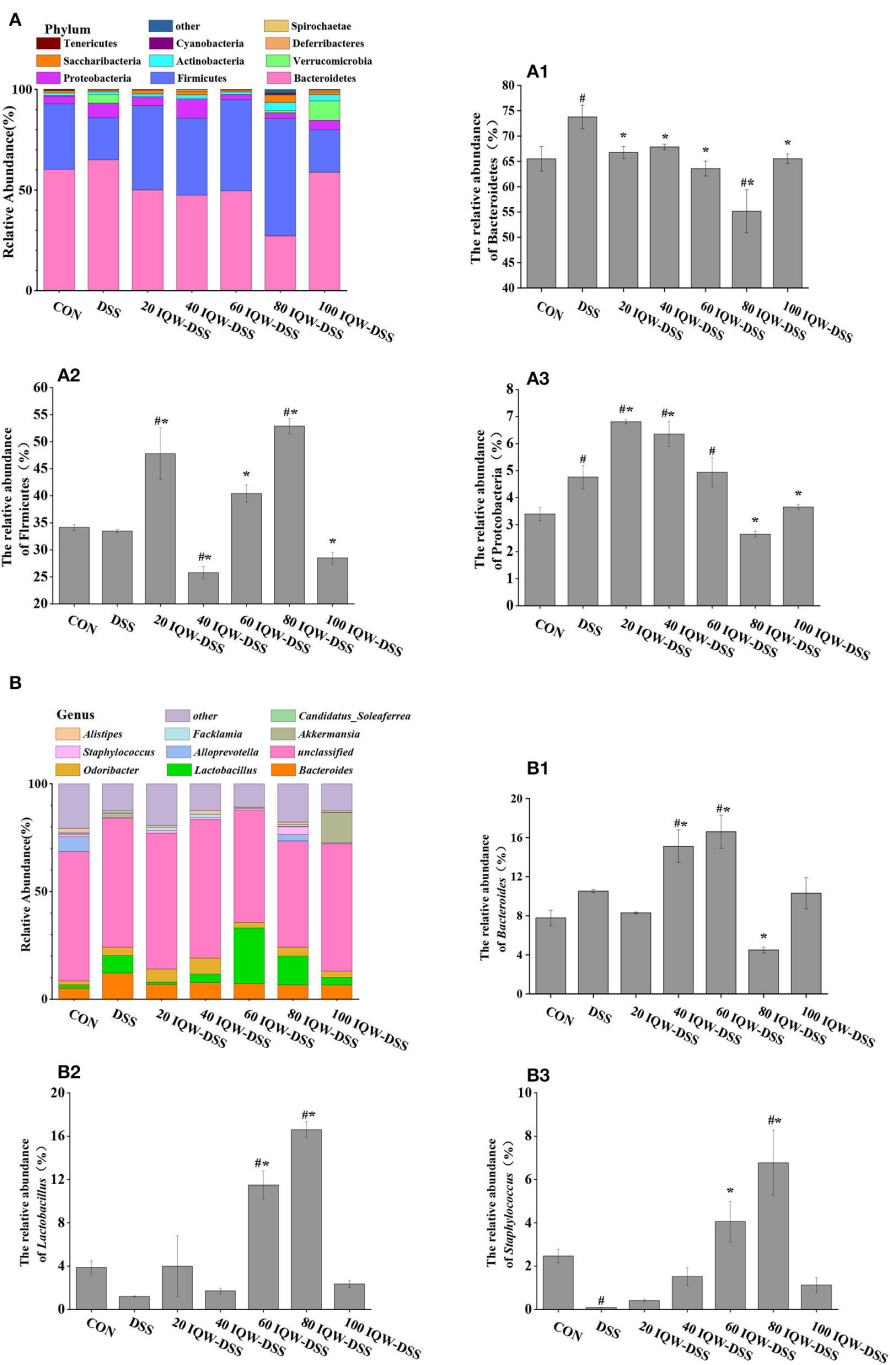
Analysis of microbial composition in the progression of colitis. **(A)** Changes in colonic microorganisms at the phylum level: **(A1)** Bacteroidetes, **(A2)** Firmicutes, and **(A3)** Proteobacteria. **(B)** Changes in colonic microorganisms at the genus level: **(B1)** *Bacteroides*, **(B2)** *Lactobacillus*, and **(B3)** *Staphylococcus*. CON: control group, DSS: mice treated with 2.5% DSS, 20 IQW-DSS: 20 μg/ml IQW and 2.5% DSS-treated mice, 40 IQW-DSS: 40 μg/ml IQW and 2.5% DSS-treated mice, 60 IQW-DSS: 60 μg/ml IQW and 2.5% DSS-treated mice, 80 IQW-DSS: 80 μg/ml IQW and 2.5% DSS-treated mice, and 100 IQW-DSS: 100 μg/ml IQW and 2.5% DSS-treated mice; *n* = 8. The results are given as mean ± SEM. ^#^*p* < 0.05 vs. the control group, *p < 0.05 vs. the DSS group.

Based on the 16S rDNA high-throughput sequencing of colonic contents analyzed with the alpha diversity index, the results showed no significant differences in alpha diversity indices among the groups (Figures 3, 4). The analysis of the microbiota at the phylum level revealed Firmicutes, Bacteroidetes, and Proteobacteria as the dominant phyla, and DSS induction increased Bacteroidetes and Proteobacteria compared to the control group. Compared with the CON group, 80 μg/ml IQW significantly decreased the abundance of Bacteroidetes (Figure 4A1), Firmicutes were significantly increased at 20 and 80 μg/ml IQW, and were significantly decreased at 40 μg/ml IQW (Figure 4A2); 20, 40, and 60μg/ml IQW significantly increased Proteobacteria abundance (Figure 4A3). Compared with the DSS group, the abundance of Bacteroidetes in the colon of mice in the 20, 60, 80, and 100 μg/ml IQW groups was significantly decreased (*p* < 0.05). Compared with the DSS group, the abundance of Firmicutes in the colon of the 20, 60, and 80 μg/ml IQW groups was significantly increased. The abundance of Firmicutes in the colon of the 40 μg/ml IQW group was significantly decreased (*p* < 0.05). Compared with the DSS group, the abundance of Proteobacteria in the colon of mice in the 20 and 40 μg/ml IQW groups was significantly increased, while in the colon of the 80 and 100 μg/ml IQW groups it was decreased considerably (*p* < 0.05) (Figure 4A).

After the analysis at the genus level, the dominant bacteria were found to be *Bacteroides, Lactobacillus*, and *Staphylococcus*, which accounted for 11-32%. In the control group, the relative abundances were 7.8% *Bacteroides*, 3.88% *Lactobacillus*, and 2.47% *Staphylococcus*. In the DSS group, the relative abundances were 10.51% *Bacteroides*, 1.2% *Lactobacillus*, and 0.08% *Staphylococcus*. In the 20 μg/ml IQW-DSS group, the relative abundances were 8.3% *Bacteroides*, 4% *Lactobacillus*, and 0.41% *Staphylococcus*. In the 40 μg/ml IQW-DSS group, the relative abundances were 15.12% *Bacteroides*, 1.72% *Lactobacillus*, and 1.52% *Staphylococcus*; In the 60 μg/ml IQW-DSS group, the relative abundances were 16.6% *Bacteroides*, 11.49% *Lactobacillus*, and 4.06% *Staphylococcus*. In the 80 μg/ml IQW-DSS group, the relative abundances were 4.5% *Bacteroides*, 16.6% *Lactobacillus*, and 6.78% *Staphylococcus*. In the 100 μg/ml IQW group, the relative abundances were 10.31% *Bacteroides*, 2.35% *Lactobacillus*, and 1.13% *Staphylococcus*. Compared with the CON group, the abundance of *Bacteroides* in the colon of the DSS group was increased significantly, and the abundance of *Staphylococcus* was decreased significantly (*p* < 0.05). Compared with the CON group, the abundances of *Lactobacillus* and *Staphylococcus* at 60 and 80 μg/ml IQW were significantly increased (Figures 4B2,B3). Compared with the DSS group, the abundance of *Bacteroides* in the colon of the 40 and 60 μg/ml IQW groups was increased significantly, while that of the 80 μg/ml IQW group was decreased significantly (*p* < 0.05). Compared with the DSS group, the abundance of *Lactobacillus* and *Staphylococcus* in the colon of the 60 and 80 μg/ml IQW groups was increased significantly (*p* < 0.05) (Figure 4B).

### Effect of different concentration gradients of egg active peptide IQW on volatile fatty acids of intestinal metabolites during the development of colitis induced by DSS

**Figure 5:**
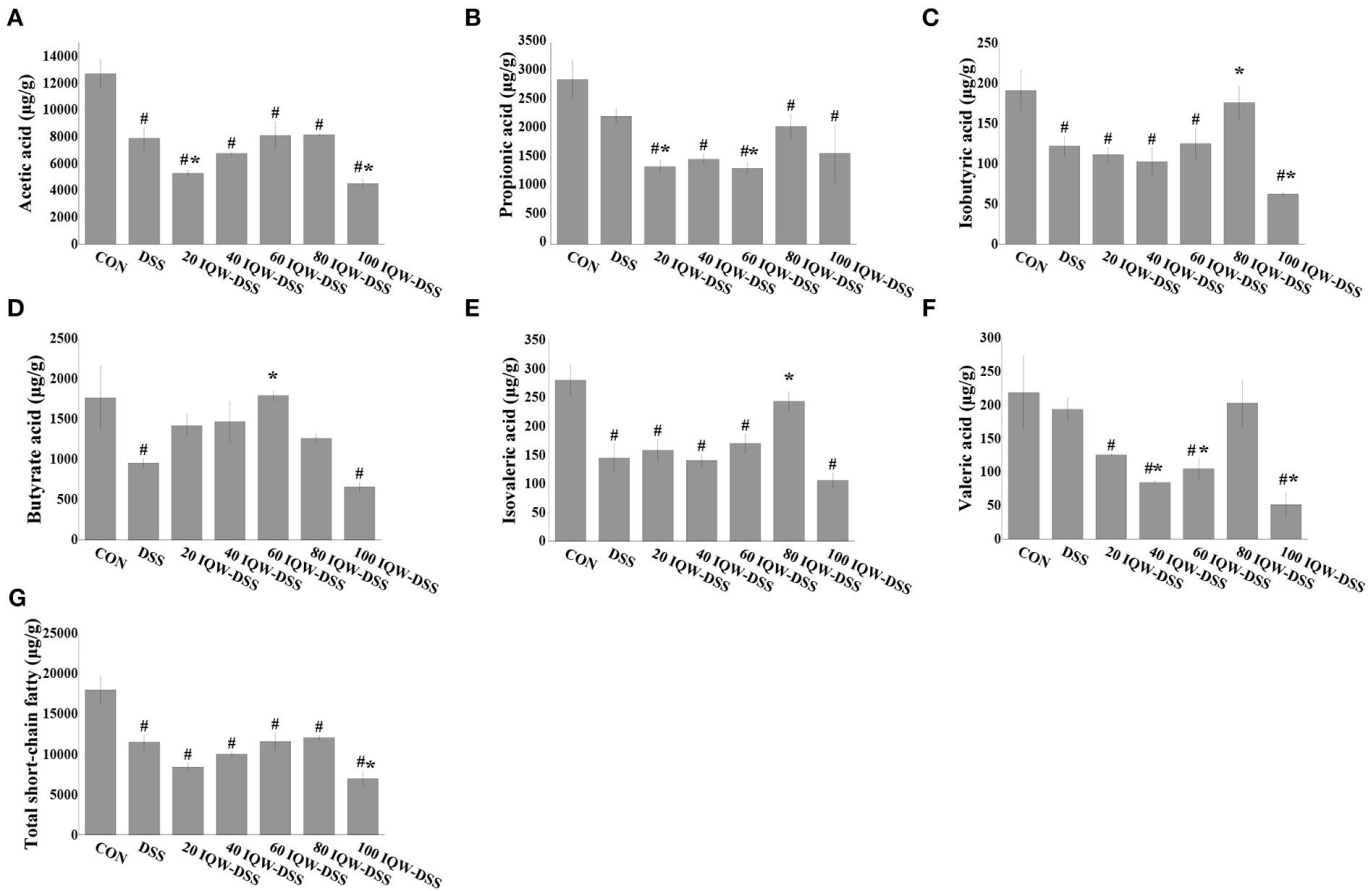
Effects of egg protein transferrin-derived peptide IQW on level of short-chain fatty acids during the development of DSS-induced colitis. **(A)** Acetic acid, **(B)** propionic acid, **(C)** isobutyric acid, **(D)** butyrate, **(E)** isovaleric acid, **(F)** valeric acid, and **(G)** total short-chain fatty acids. CON: control group, DSS: mice treated with 2.5% DSS, 20 IQW-DSS: 20 μg/ml IQW and 2.5% DSS-treated mice, 40 IQW-DSS: 40 μg/ml IQW and 2.5% DSS-treated mice, 60 IQW-DSS: 60 μg/ml IQW and 2.5% DSS-treated mice, 80 IQW-DSS: 80 μg/ml IQW and 2.5% DSS-treated mice, and 100 IQW-DSS: 100 μg/ml IQW and 2.5% DSS-treated mice; *n* = 8. The results are given as mean ± SEM. ^#^*p* < 0.05 vs. the control group, *p < 0.05 vs. the DSS group.

The effects of egg protein transferrin-derived peptide IQW on the level of short-chain fatty acids during the development of DSS-induced colitis are shown in Figure 5. Compared with the CON group, the contents of acetic acid, isobutyric acid, butyric acid, isovalerate, and total SCFAs in the feces of mice in the DSS group were significantly decreased (*p* < 0.05) (Figures 5A,C–E,G). Compared with the CON group, the levels of total short-chain fatty acids, acetic acid, propionic acid, and isovaleric acid were significantly reduced in the IQW-DSS treatment groups at different concentrations (Figures 5A,B,E,G). The levels of isobutyric acid and valeric acid were significantly reduced in the 20, 40, 60, and 100 μg/ml IQW groups (Figures 5C,F), and the level of butyric acid was significantly reduced in the 100 μg/ml IQW-treated group (Figure 5D). Compared with the DSS group, the acetic acid content in the feces of mice in the 20 and 100 μg/ml IQW groups was significantly decreased, and the propionate content in the feces of mice in the 20 and 60 μg/ml IQW groups was considerably reduced (*p* < 0.05) (Figures 5A,B). Compared with the DSS group, the contents of isobutyric acid and isovaleric acid in the feces of the 80 μg/ml IQW group were significantly increased, while those of the 100 μg/ml IQW group were significantly decreased (*p* < 0.05) (Figures 5C,E). Compared with the DSS group, the butyric acid in the feces of mice in the 60 μg/ml IQW group was increased (*p* < 0.05) (Figure 5D). Compared with the DSS group, the valeric acid in the feces of mice in the 40, 60, and 100 μg/ml IQW groups was decreased significantly (*p* < 0.05) (Figure 5F). Compared with the DSS group, the total SCFAs in mice feces in the 20 and 100 μg/ml IQW groups was significantly decreased (*p* < 0.05) (Figure 5G).

### Effect of intestinal microflora on volatile fatty acid levels in intestinal metabolites

**Figure 6:**
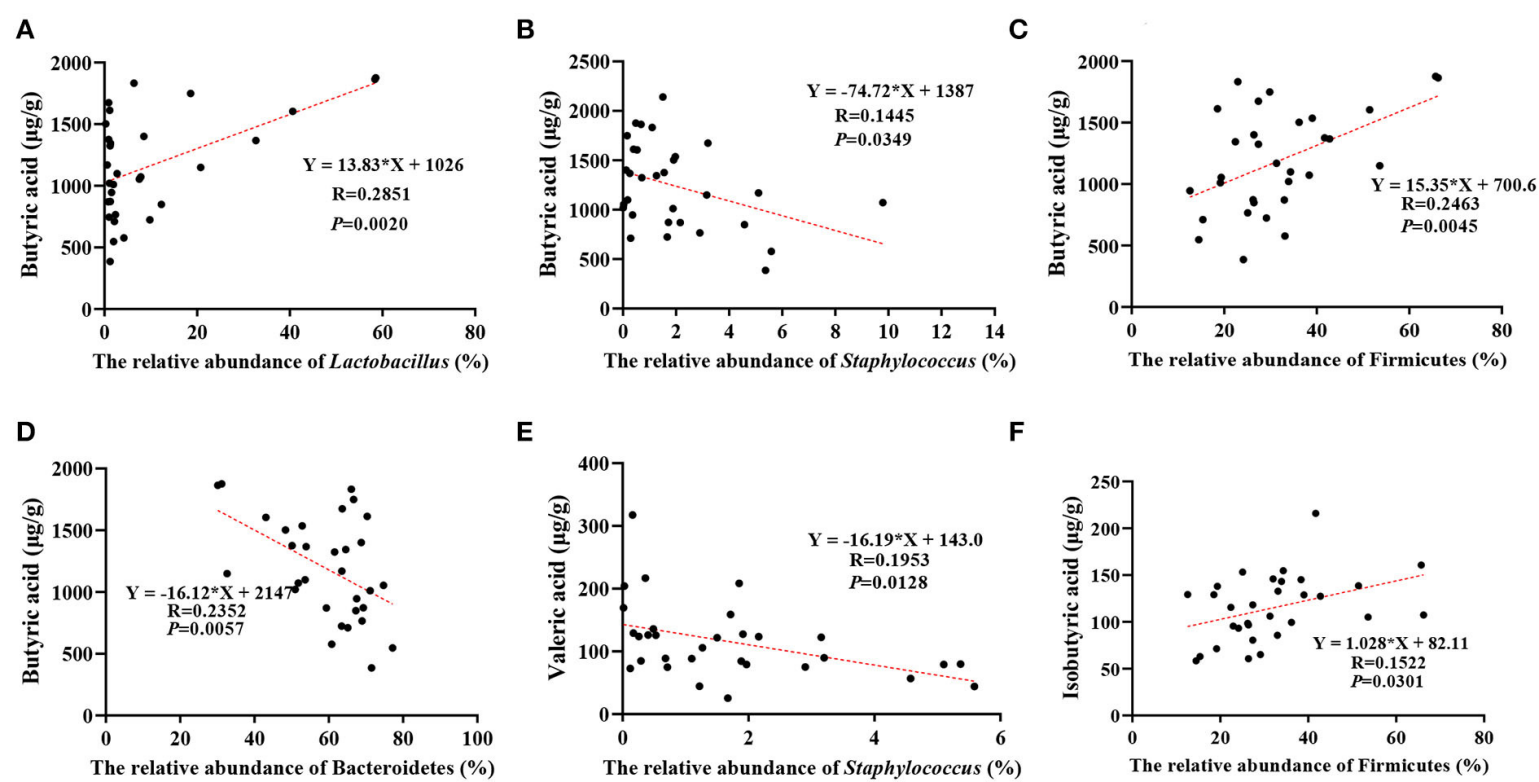
Correlation between different microorganisms and different short-chain fatty acids in the development of DSS-induced inflammation. **(A)** Effect of relative abundance of *Lactobacillus* on butyrate acid, **(B)** effect of relative abundance of *Staphylococcus* on butyrate acid, **(C)** effect of relative abundance of Firmicutes on butyrate acid, **(D)** effect of relative abundance of Bacteroidetes on butyrate acid, **(E)** effect of relative abundance of *Staphylococcus* on valeric acid, and **(F)** effect of relative abundance of Firmicutes on Isobutyric acid. The results are given as mean ± SEM (*n* = 31), *p* < 0.05.

The correlation analysis of microflora and volatile fatty acids in the gut revealed that the butyrate levels were correlated positively with the abundance of *Lactobacillus* (*p* = 0.002) and Firmicutes (*p* = 0.0045) and negatively with the abundance of *Staphylococcus* (*p* = 0.0349) and Bacteroidetes (*p* = 0.0057) (Figures 6A–D), the valerate levels were negatively correlated with *Staphylococcus* abundance (*p* = 0.0128) (Figure 6E), and the isobutyrate levels were positively correlated with the abundance of Firmicutes (*p* = 0.0301) (Figure 6F).

## Discussion

IBD is a multifactorial disease with multiple complications, which make the diagnosis and treatment of IBD a major challenge. The DSS-induced mouse colitis model has similar clinical symptoms of colitis, such as weight loss, metabolic disorders, and intestinal flora disorders (20–22). In this study, the effects and possible mechanisms for the relief of colitis were investigated by administering different concentration gradients of IQW in the context of DSS induction.

Ovotransferrin can bind to metal ions to enhance its antioxidant properties in addition to its antibacterial activity (23). In the present study, we found that the administration of 2.5% DSS induced weight loss, bloody stools, shortened colon length, and impaired colon histomorphology in mice, and that IQW treatment alleviated the negative effects of DSS and improved weight loss, shortened colon length, and colonic erosion. In addition, in the presence of DSS, the concentration of 60 μg/ml IQW significantly alleviated DSS-induced colitis in the mice. This may be related to amino acids that comprise IQW (15). Tryptophan is involved in the kynurenine, indole, and 5-hydroxytryptamine pathways, and it is essential for regulation of IBD by acting on immune cells, pro- or anti-inflammatory cytokines, and the microbial composition of the gut (24). Glutamine, a critical respiratory substrate for intestinal cells, maintains and restores intestinal function during enteritis by inducing MKP-1 kinase (25).

Progression of IBD is associated with microbial ecological dysbiosis, so controlling gut microbial dysbiosis can be conducted to treat IBD (26). Changes in the gut microbiome are responsible for many inflammatory diseases, including colitis, and when the microbiome is dysregulated, it induces inflammatory responses and immune-induced diseases (27). *Bacteroides, Lactobacillus, Prevotella*, and *Ruminococcus* are florae associated with IBD (28). The microbial diversity of the gut and its functions are altered in patients with IBD compared to the healthy gut, and examples include Firmicutes, Bacteroidetes, and Proteobacteria. There is evidence that the abundance of anti-inflammatory florae, such as *Bifidobacterium* and *Bacteroides*, is decreased during inflammation (7, 29). In this test, the alpha diversity index of microorganisms has no significant change, which may be due to the poor homogeneity among the mice. However, by the analysis of the dominant species at the phylum and genus levels, it was found that compared with the DSS-induced group, the IQW treatment group had increased abundance of Firmicutes and decreased abundance of Bacteroidetes at the phylum level; At the genus level, IQW treatment showed an increasing trend for *Lactobacillus* and *Staphylococcus* and a decreasing trend for *Bacteroides*. Liu et al. found in their research with an IBD model that the level of Bacteroides was restored during IQW treatment (17). Liu et al. also found that IQW reversed the change in intestinal dominant flora abundance in the treatment of ETEC-induced inflammation in a model (30). Ma Yong et al. (31). reported that IQW treatment could restore the levels of Firmicutes, Bacteroidetes and *Lactobacillus*, but that it had no significant impact on intestinal microbial diversity, which was consistent with the results of this test. These results suggest that IQW treatment can reprogram the intestinal microbial community. To some extent, it can restore the intestinal flora disorder induced by inflammation.

In addition, IQW can improve colitis through microbial metabolic pathways. SCFAs, produced by digestion of complex dietary fiber by microorganisms, are important energy and signal molecules that affect the physiological health of the host and have the characteristics of resisting inflammation, protecting the intestinal barrier, and maintaining intestinal homeostasis (6, 11). The results of this study showed that although there was no significant effect of IQW therapy on total SCFAs under DSS induction, the levels of SCFAs were lowered in the DSS group, 60 μg/ml IQW increased butyric acid and reduced valeric acid levels, and 80 μg/ml IQW increased the branched-chain fatty acids isobutyric and isovaleric acids. This may be related to changes in microbial diversity. For example, Firmicutes are the main microbial species producing butyrate in the colon, and the lactic acid produced by *Lactobacillus* can promote the production of butyrate (32, 33), while its abundance changes after IQW treatment. Thus, IQW treatment of colitis can be achieved by regulating microorganisms and their metabolism. However, branched-chain fatty acids are generally considered to be harmful to the colon, as the fermentation of proteins to produce branched-chain fatty acids is accompanied by fermentation products, such as ammonia, phenol, p-formic acid, and hydrogen sulfide, which may affect the epithelial cells of the colon (34). Moreover, studies have proved that isovaleric acid can directly affect the longitudinal smooth muscle cells of the colon of mice, activate the protein kinase A (PKA) signal, and cause relaxation of the smooth muscle of the colon (35). It is therefore particularly important that the appropriate concentration is chosen for the treatment of colitis with IQW.

We suggest that IQW can alleviate DSS-induced weight loss, fecal occult blood, and histological damage. At the same time, egg active peptide IQW can also regulate the intestinal microbial community structure in mice, alleviate DSS-induced colon microbial disorder, and increase the abundance of probiotics, such as *Lactobacillus*. Overall, egg-active peptide IQW can alleviate DSS-induced colitis.

## Data availability statement

The datasets presented in this study can be found in online repositories. The name of the repository and accession number can be found below: National Center for Biotechnology Information (NCBI) BioProject, https://www.ncbi.nlm.nih.gov/bioproject/, PRJNA832929.

## Ethics statement

The animal study was reviewed and approved by the Hunan Agricultural University Animal Care and Use Committee.

## Author contributions

YC: writing (original draft preparation). SD: study protocol. LJ, SW, and XY: index detection. HJ and JF: writing (review and editing). All authors contributed to manuscript revision, and read and approved the submitted version.

## Funding

This research was supported by the National Key Research and Development Program of China (2021YFD1300205-2), the National Natural Science Foundation of China (81902297 and 81900441), Natural Science Foundation of Zhejiang Province (LQ19H020002), Hunan Provincial Science and Technology Department (2020NK2004, 2019TP2004, 2018WK4025, and 2020ZL2004), Local Science and Technology Development Project Guided by The Central Government (YDZX20184300002303 and 2018CT5002), Scientific Research Fund of Hunan Provincial Education Department (2020JGYB112), and Double FirstClass Construction Project of Hunan Agricultural University (SYL201802003, YB2018007, CX20190497, and CX20190524).

## Conflict of interest

The authors declare that the research was conducted in the absence of any commercial or financial relationships that could be construed as a potential conflict of interest.

## Publisher's note

All claims expressed in this article are solely those of the authors and do not necessarily represent those of their affiliated organizations, or those of the publisher, the editors and the reviewers. Any product that may be evaluated in this article, or claim that may be made by its manufacturer, is not guaranteed or endorsed by the publisher.
